# Nigerian indigenous hens show more discomfort-related behavior with visual separation than physical separation from their chicks: An exploratory study

**DOI:** 10.3389/fvets.2022.978848

**Published:** 2022-11-03

**Authors:** Oluwaseun S. Iyasere, Olawale P. Olajumoke, Samuel O. Durosaro, O. E. Oke, Oluwabukunmi O. Famosaya, Kolade M. Oliyide, Victor J. Oyeniran

**Affiliations:** ^1^Department of Animal Physiology, Federal University of Agriculture, Abeokuta, Nigeria; ^2^Albrecht Daniel Thaer-Institut für Agrar- und Gartenbauwissenschaften Tierhaltungssysteme und Ethologie, Humboldt University, Berlin, Germany; ^3^Department of Animal Breeding and Genetics, Federal University of Agriculture, Abeokuta, Nigeria; ^4^Department of Animal Sciences, Purdue University, West Lafayette, IN, United States

**Keywords:** behavior, maternal care, Nigerian indigenous chickens, separation types, welfare, pacing

## Abstract

The Nigerian indigenous hens exhibit their full natural behavior repertoires, including maternal care. The strong maternal bond between the hen and her chicks is established prior to hatching. Maternal care of chickens is essential for both exotic and indigenous chickens. This study compared the behaviors of six hen-chick pairs in a physical (PHY) and visual (VIS) separation test for 10 min. All the six hen-chick pairs were subjected to PHY separation on the 8^th^ day of post-hatch and a VIS separation on the 12^th^ day of post-hatch. The PHY separation involved the use of a wire mesh to separate the hen from her chicks, while the VIS separation involved the use of a trampoline to separate the hen from her chicks. The hen's behavior was recorded during the 10-min separation period. Behaviors recorded included sitting, body shaking, pecking, movements toward the chicks, jumping, pacing, defecation, movements away from the chicks, and preening. We further grouped these nine behaviors into two categories: discomfort-related (pacing, movement toward chicks, body shaking, defecation, and jumping) and comfort-related (sitting, pecking, preening, and movement away from the chicks) behaviors. Before and after each separation, the hens were gently restrained, and a drop of blood was sampled from the wing vein to determine the blood glucose level. Their heart rate and eye temperature were also measured. A two-related samples test (Wilcoxon) was used to compare the behavior of the hens when subjected to the PHY and VIS separation. Eight out of the nine behaviors monitored did not differ between the separation types. However, the frequency of pacing by the hens was greater (z = −2.201, *P* = 0.028) in the VIS separation than in the PHY separation. Also, discomfort-related behavior was greater (*t*_(5)_ = −2.717, *P* = 0.042) during the VIS separation than the PHY separation. Comfort-related behavior did not differ between the separation types. The change in eye temperature, heart rate, and blood glucose was similar in the two separation types. In conclusion, Nigerian indigenous hens displayed more discomfort-related behavior to the VIS separation from their chicks, but this was not associated with physiological responses indicative of stress.

## Introduction

The Nigerian indigenous chicken is the most common poultry species found in the rural areas of Nigeria (1). They are commonly reared under an extensive or semi-intensive system (2). These chickens are raised for cultural and socio-economic purposes. The birds are usually provided minimal nutrition, medication, and shelter, which can compromise their welfare. In a scavenging system, the hen and her chicks may be separated temporarily (short-term) or permanently (long-term). During scavenging, a predator may kill the chicks, chicks may get lost when trapped by weeds, ropes, or threads, and a physical barrier such as a fence may not allow the hen to find her chicks. Also, the owner of the chickens may decide to wean the chicks early and sell them for financial reasons. The consequences of these circumstances on the welfare of the hens and the chicks are unknown but there is evidence that hens respond to their chicks in distress. Mother hens showed context-dependent behavioral responses. They also showed physiological responses such as changes in eye and comb temperature (a measure of stress-induced hyperthermia), heart rate, and heart rate variability (a measure of the activation of the sympathetic and parasympathetic nervous systems, respectively) when physically separated from their chicks. When the chicks are separated without any aversive stimulus, the hen showed no physiological changes in terms of heart rate and eye temperature but the mother hen responded to her chicks being puffed with a drop in eye temperature and an increase in heart rate (3).

The welfare of an animal is good when the animal is allowed to perform its natural behaviors (4). Although not all natural behaviors are beneficial, maternal behavior is one of the most important natural behaviors of the indigenous chickens which has helped them survive and maintain their population for several years. Maternal behavior in chickens includes nesting, egg-laying, brooding, and post-hatch care of chicks (5). Broodiness is often considered an uneconomical trait because the hen stops laying (6). This trait has been selected against in most commercial laying hens. However, most indigenous breeds remain genetically unselected for increased egg production and still exhibit broodiness, making it possible for them to incubate their eggs and hatch their chicks by themselves. The Nigerian indigenous hens spend 88–93% of their time sitting on the eggs and 0.06–0.11% on feeding and drinking during the brooding period (7). Broodiness is also associated with changes in breast temperature and blood glucose levels in Nigerian indigenous hens (7). In a comparative study, the Yoruba ecotype of the Nigerian indigenous hens spent more time sitting on the eggs during brooding than the Fulani ecotype hens (8).

Bonding in chickens starts a few days before hatching, at a time when the hen and the developing embryo begin to communicate through vocalization (9). This pre-hatching communication enables the chicks to recognize the voice of their mothers after hatching. After hatching, the hen serves as the role model for her chicks by providing warmth and protection. The hen teaches her chicks how and where to forage and escape from predators using a variety of calls (9). The Thai native hens protect their chicks by being vigilant, aggressive, and emitting an alarm call (10). Maternal care of chickens is essential and cannot be underestimated. Also, maternal deprivation of chicks has welfare consequences (9). Chicks reared without mothers are highly fearful, aggressive, and displayed higher feather pecking and cannibalism (11–15).

Chickens are a precocial species, which means that the chicks can survive on their own in an artificial environment after hatching. However, in the natural environment, day-old chicks cannot survive without their mother's care because they cannot regulate their body temperature and escape from predators. The external temperature experienced by red junglefowl chicks in the wild ranges from 19–28°C, a temperature range that the chicks may find difficult to cope with until they are about 10 days old (16). Prior to this age, the mother hen provides the needed warmth (16).

Mother hens permanently separated from their chicks after 5 to 10 days post-hatch, resumed egg-laying earlier, but the chicks had a low survival rate (6). Although Amin et al. (6) did not report the behavior of the hens and chicks during the separation, the abrupt weaning could be stressful for both the hen and her chicks. Indigenous chickens are mostly reared by rural farmers in most developing countries. The hens are used to produce the next generation of the chicken flock by allowing them to incubate and care for the chicks. There seems to be no information on the best approach to artificially wean chicks from the hens. Some farmers may be patient enough for the hen to wean the chicks naturally (which could range from 5–12 weeks post-hatch), but farmers that use this hen as a “natural hatcher” prefer to wean the chicks immediately after hatching and place another set of eggs under the hen to incubate for another 21 days. Using the broody hen to hatch two batches of chicks consecutively may have a detrimental effect on the hen's welfare and need further investigation. Also, there is a need to develop a more welfare-friendly method of weaning chicks from their mothers than the conventional sudden separation.

After hatching, hens display maternal aggression, which is necessary to protect their chicks from environmental threats such as humans and predators. The survivability of the chicks in the natural environment is highly dependent on how protective the mother hen is. Since these chickens are reared under scavenging systems, it is of utmost importance to identify and select hens with a high level of maternal aggression, as this would ensure better chick survivability. So, in this exploratory study, we compared the behaviors of six Nigerian indigenous hens when separated physically or visually from their chicks. The use of less invasive means of assessing stress, such as infra-red thermography and heart rate monitors, serves as a refinement of the procedures for assessing animal welfare. Glucocorticoids are known to be stress hormones, but they have several limitations, some of which are the need to sample blood within a short time (<3 mins), handling the animal can trigger the release of glucocorticoids and an increase in glucocorticoids does not indicate whether the subject is experiencing a positive or negative valence (17). We hypothesized that hens would find the two separation types different and therefore display discomfort-related behaviors coupled with stress responses to the separation type that prevents them from having more contact with their chicks. The implications of these separation types are discussed with respect to the management and animal welfare purposes.

## Materials and methods

### Ethical statement

The procedure for the experiment was approved by the Animal Care and Use Committee of the College of Animal Science and Livestock Production, Federal University of Agriculture, Abeokuta, Ogun State, Nigeria. All birds used in the experiment were provided with proper care and management, and were not exposed to unnecessary discomfort.

### Experimental site

The experiment was conducted at the Poultry Unit of the Federal University of Agriculture, Abeokuta, Nigeria. The experimental site lies on latitude 7°10'N and longitude 3°2'E. It is located 76 m above sea level, in the tropical rainforest vegetation zone, and has a mean temperature of 28.5°C.

### Experimental procedure

The birds used in this study were sourced from an existing flock of sexually mature Nigerian indigenous chickens (30 hens and three cocks) of the Yoruba ecotype. The average weight of the cocks and hens was 1250 ± 90.5 g and 850 ± 50.5 g, respectively. The birds were housed in deep litter pens with a mating ratio of 10 hens to one cock per pen (975g/m^2^). The birds were fed layer mash (16.5% CP, 2725–2980 Kcal/kg metabolizable energy, 5% fat/oil, 6% crude fiber, 3.60% calcium, 0.45% available phosphorus, 0.80% lysine, 0.34% calcium, and 0.30% salt) at the rate of 120 g/bird/day (the recommended quantity for a laying hen to prevent fat accumulation that can affect laying) and fresh water was provided *ad libitum*. The pens were furnished with wooden ladder perches (each consisting of three tiers; the lower tier at 20 cm, the middle tier at 50 cm, and the upper tier at 90 cm above the ground) for the birds to roost at night and nest boxes for the hens to perform their natural egg-laying behavior. The health of the birds was checked daily. Eggs laid in the nest boxes were left to encourage broodiness. Any hen that became broody (as demonstrated by continuous sitting on the eggs for three consecutive days) was separated into a brooding pen and provided with 10 fresh eggs (eggs picked from the remaining flock) to incubate and feed and water *ad libitum*. The expected hatching date from the day the hen was separated and provided with the fresh eggs to incubate was noted for each hen. The poultry house was open-sided, so birds were exposed to natural daylight (~12L:12D) and daily fluctuations in temperature and humidity.

We intended to have data from as many hens that became broody as possible. However, there were cases of two broody hens for which we could not get data. The first hen left its nest on the 17^th^ day of brooding and did not return to sit on the eggs. We later found out that none of the eggs was fertile. The hen was probably sensitive enough to detect this, as the hen must have expected that there should be a pre-hatching communication between her and the developing embryos at that stage of incubation. The second hen died during brooding; the cause was unknown to us.

The six mother hen-chick pairs were undisturbed for the first 7 days post-hatch. Water was provided in a bell drinker (diameter = 21.5 cm, depth = 20.3 cm) and feed was provided in round plastic tray feeders (diameter = 20.0 cm). Chick mash (21% CP, 3000 Kcal/kg metabolizable energy) was provided to the hen and her chicks after hatching (as it was not possible to feed them separately). All six hen-chick pairs (each hen-chick pair served as a replicate) experienced both separation types; physical separation on day 8 and visual separation on day 12 post-hatch. Each hen-chick pair was tested once on each of these days for a 10-min period.

### Physical separation test

**Figure 1:**
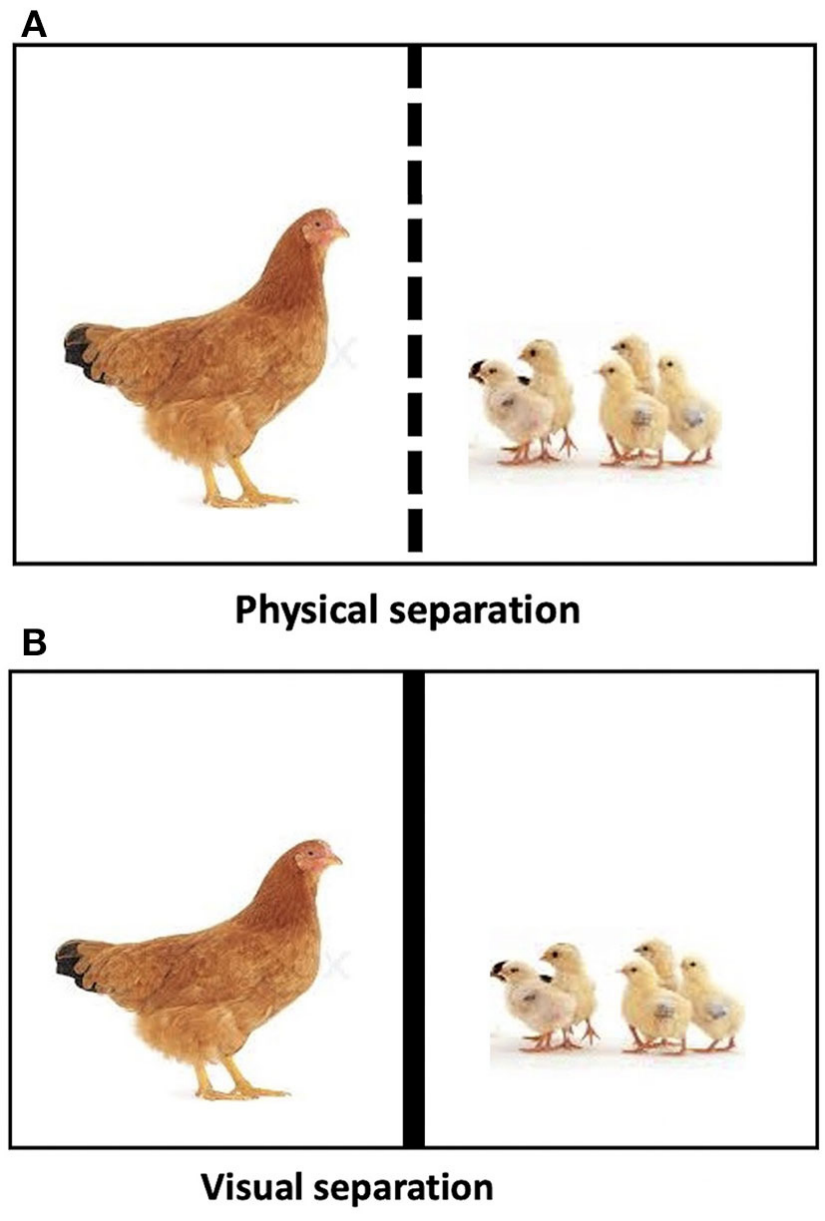
**(A)** Hen-chicks' pair subjected to physical separation in a test arena. **(B)** Hen-chicks' pair subjected to visual separation in a test arena.

On the 8^th^ day of post-hatch, each hen-chick pair was placed in a test arena separated by a wire mesh partition, which allowed visual and auditory contact between the mother hen and her chicks (Figure 1A). The hen's behaviors were recorded using a digital camera (Fujifilm S2950, made in China) during a 10-min physical separation. The frequency of behaviors, such as movements away from chicks (AFC), movements toward the chicks (TC), defecation, preening, sitting, body shaking, pecking, jumping, and pacing was recorded. A description of the behaviors is presented in Table 1.

**Table 1:** Ethogram of mother hen behavior monitored during visual and physical separation.

**Behavioral category**	**Description**
Movement away from chicks (AFC)	The hen moves away from the barrier between her and her chicks
Movement toward chicks (TC)	The hen moves closer to the barrier between her and her chicks
Defecation	Excretion of feces by the hen in the test arena
Preening	The hen uses its beak to arrange its feathers
Sitting	Hen lying down on her chest in the test arena
Body shaking	The hen shakes and ruffles her feathers
Pecking	The hen pecking on wooden materials in the test arena
Jumping	The hen jumps to escape from the test arena to reunite with her chicks
Pacing	The hen moving to and fro in the test arena without rest

### Visual separation test

On the 12^th^ day of post-hatch, the same test arena used for the physical separation test was used, but instead of having a wire mesh separating the hen from her chicks as in the physical separation, a purple trampoline was securely attached to the wire mesh partition between the hen and her chicks, thus allowing only auditory but no visual contact between the mother hen and her chicks (Figure 1B). The visual separation lasted for 10 min, and the behaviors of the hens were recorded.

The nine behaviors were further grouped into two categories: discomfort-related (pacing, movement toward chicks, body shaking, defecation, and jumping) and comfort-related (sitting, pecking, preening, and movement away from the chicks) behaviors.

### Physiological data collection

Before and after each separation type, physiological parameters such as heart rate, eye temperature, and blood glucose level were measured. The heart rate of the hens was measured with a stethoscope placed on the chest region of the hen and the number of beats per 15 s was counted and multiplied by four to give the number of beats per minute. The eye temperature of the hens was measured using an infrared thermometer (Model: IT-122, accuracy ± 0.2°C, made in China) pointed about 2 cm away from the eye of the hen. Finally, a drop of blood was sampled from the wing vein onto a glucose strip, which was immediately inserted into an ACCU-CHEK active glucose meter (manufactured by ROCHE Mannheim, Germany) to determine the blood glucose level. These physiological parameters were taken within 2 min of restraining the hen.

### Statistical analysis

The individual differences between the six hen-chick pairs during the two separation types (physical test on day 8 and visual test on day 12 post-hatch) were analyzed using descriptive statistics (bar charts). We compared each of the nine behaviors monitored from the hens in these two separation types using a two-related samples test (Wilcoxon). Similarly, discomfort-related and comfort-related behaviors displayed by each hen in the two separation types were compared using paired sample *t*-test. The change (after separation minus before separation) in the eye temperature, heart rate, and blood glucose of the six hens during the two separation types were also compared using a paired sample *t*-test. All statistical procedures were undertaken using the IBM SPSS statistical software (Version 23).

## Results

**Figure 2:**
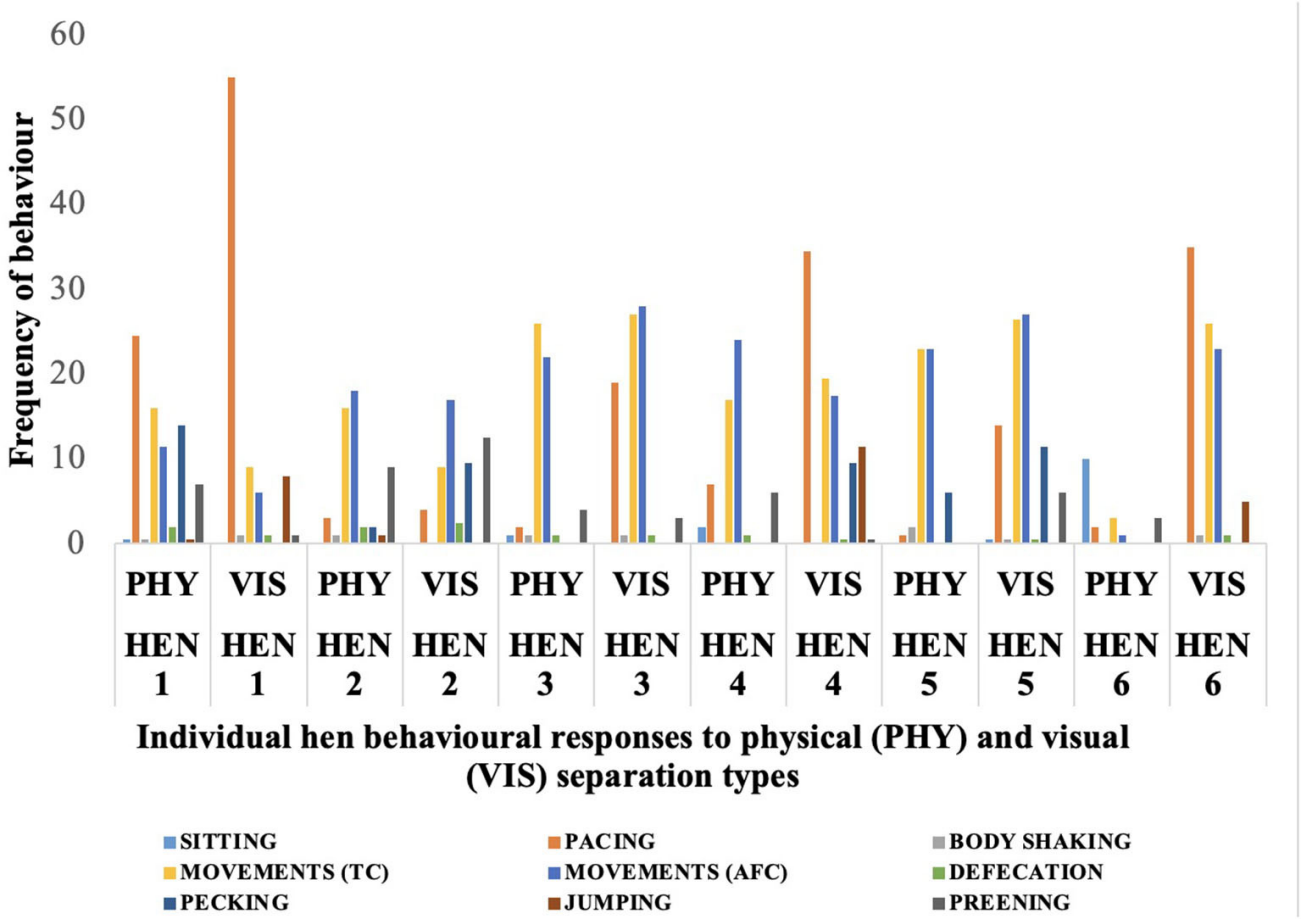
Frequency of behaviors exhibited by each of the six hens during the physical (PHY) and visual (VIS) separation tests for 10-minutes from their chicks. 1-6 represent the individual hen numbers. TC, toward chicks; AFC, away from chicks.

**Figure 3:**
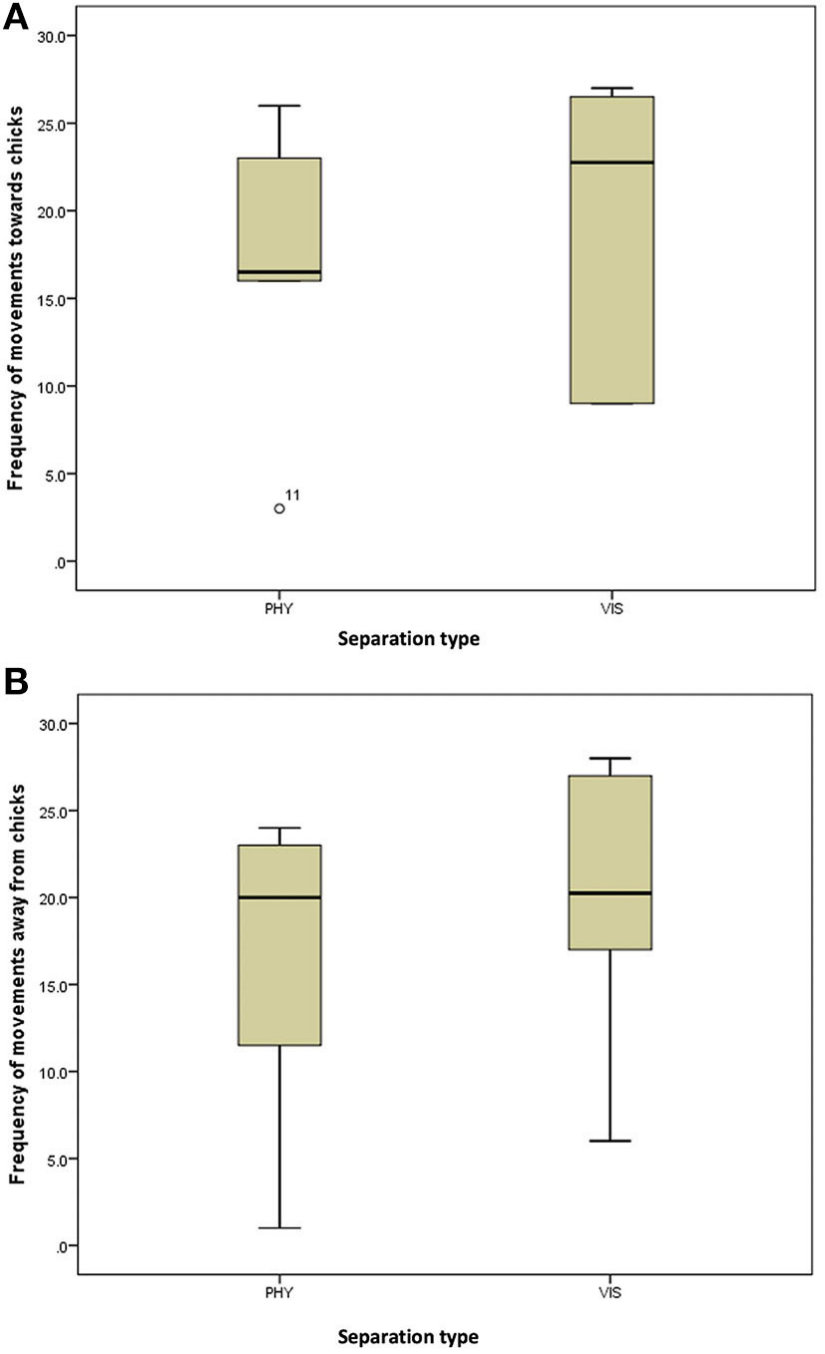
**(A)** Frequency of movement of the mother hens toward their chicks when subjected to 10-min physical (PHY) or visual (VIS) separation. **(B)** Frequency of movement of the mother hens away from their chicks when subjected to 10-min physical (PHY) or visual (VIS) separation.

**Figure 4:**
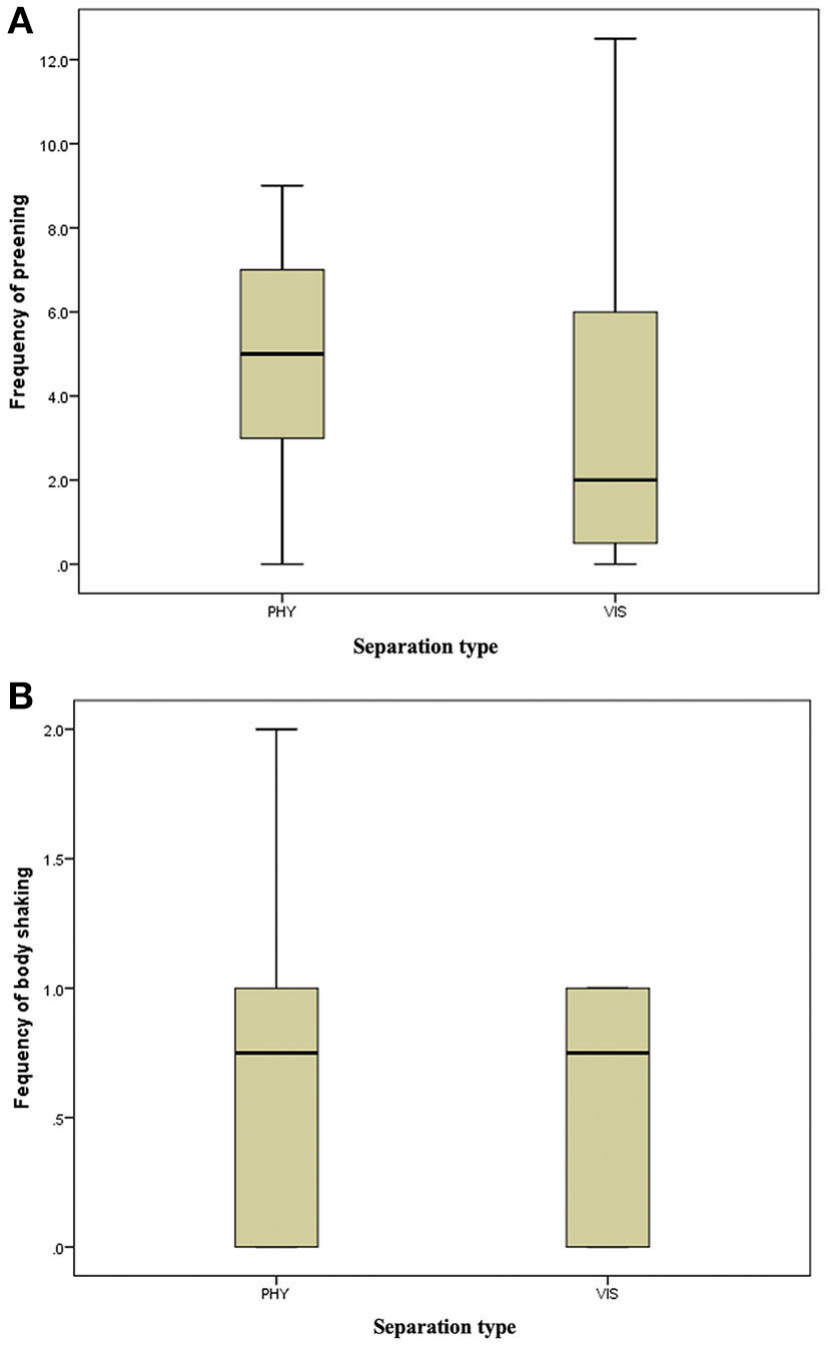
**(A)** Frequency of preening of the mother hens when subjected to 10-min physical (PHY) or visual (VIS) separation from their chicks. **(B)** Frequency of body shaking of the mother hens when subjected to 10-min physical (PHY) or visual (VIS) separation from their chicks.

**Figure 5:**
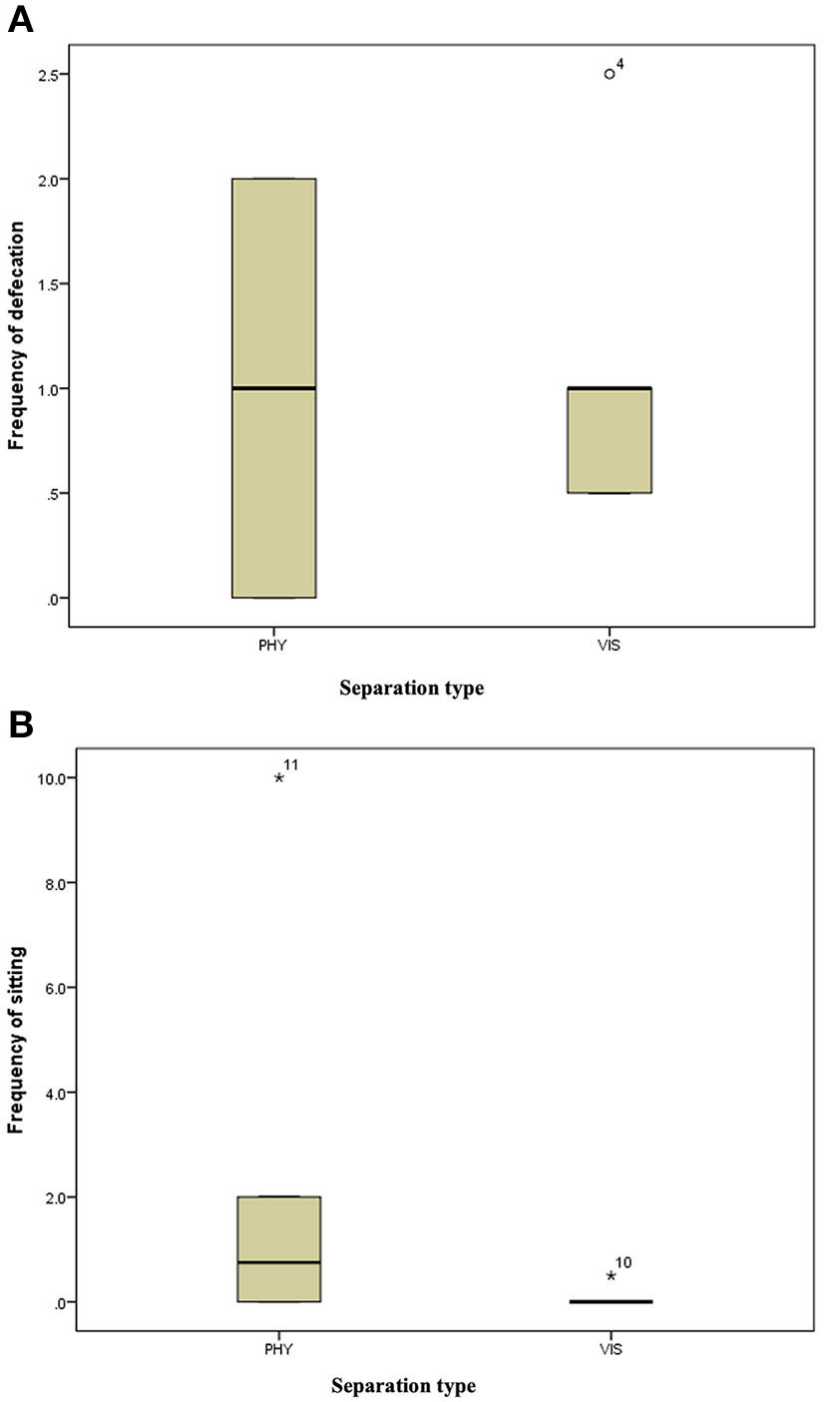
**(A)** Frequency of defecation of the mother hens when subjected to 10-min physical (PHY) or visual (VIS) separation from their chicks. **(B)** Frequency of sitting of the mother hens when subjected to 10-min physical (PHY) or visual (VIS) separation from their chicks.

**Figure 6:**
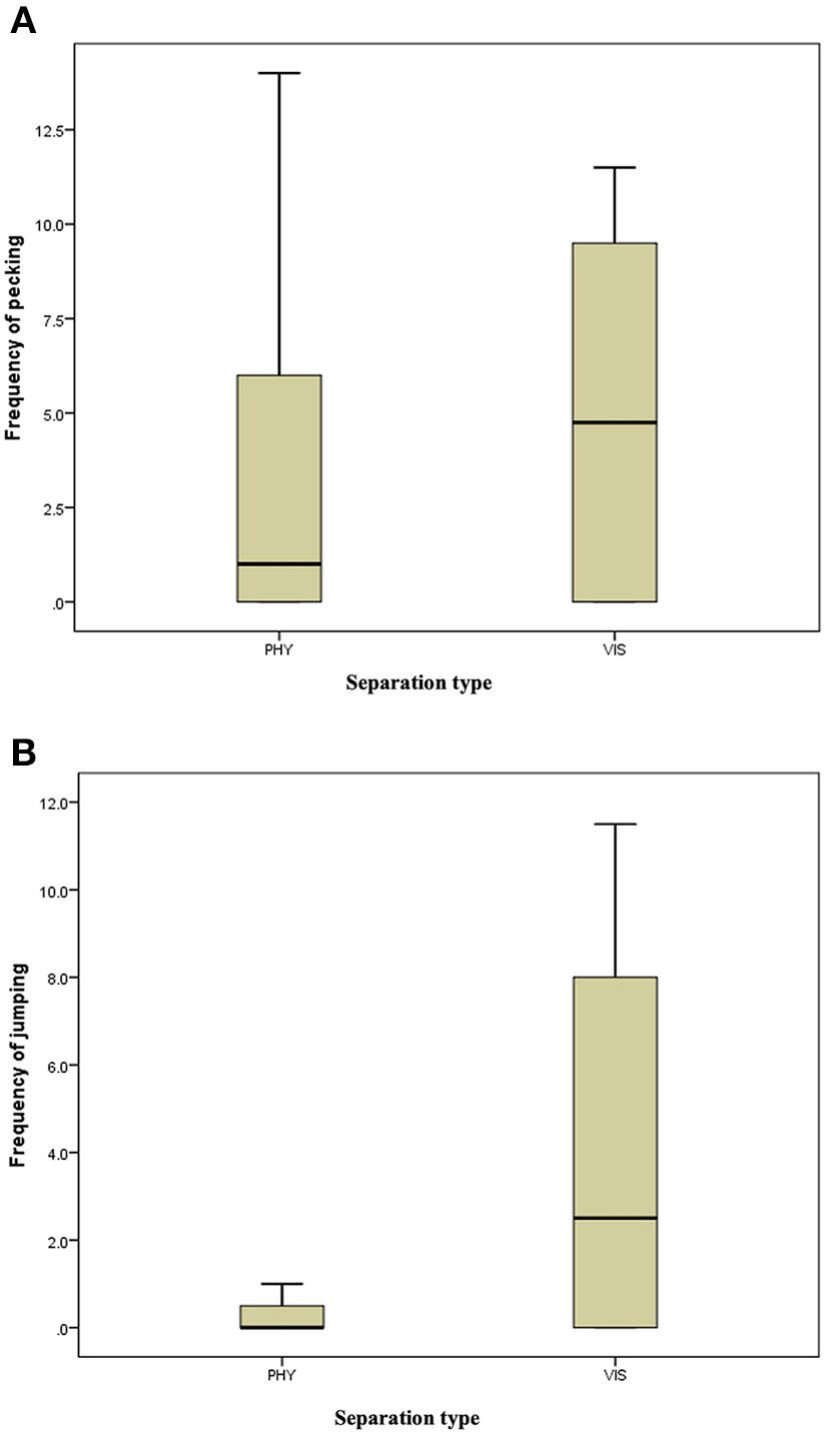
**(A)** Frequency of pecking of the mother hens when subjected to 10-minute physical (PHY) or visual (VIS) separation from their chicks. **(B)** Frequency of jumping of the mother hens when subjected to 10-minute physical (PHY) or visual (VIS) separation from their chicks.

**Figure 7:**
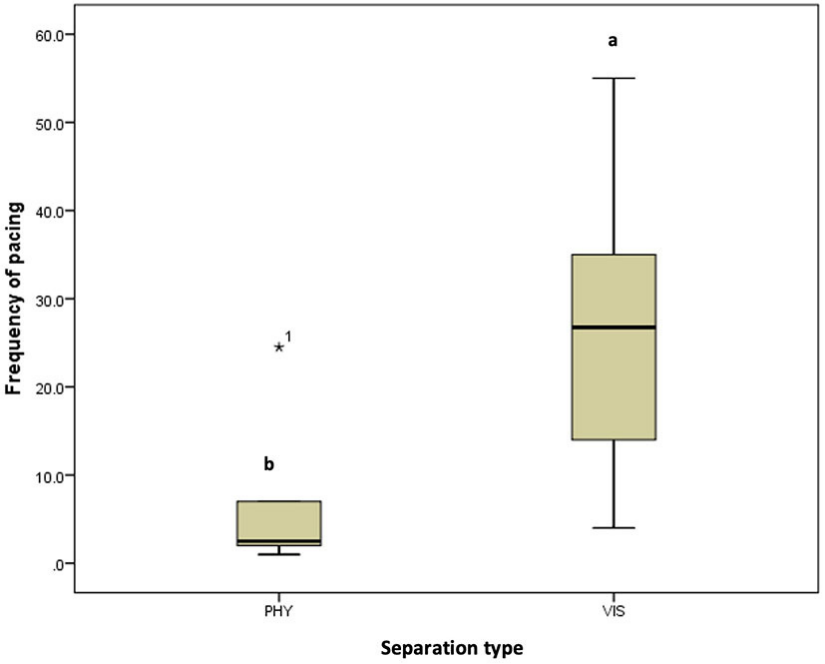
Frequency of pacing of the mother hens when subjected to 10-minute physical (PHY) or visual (VIS) separation from their chicks.^ab^ Means differ at *P* < 0.05.

Behavioral responses of individual hens to the physical and visual separation from their chicks are shown in Figure 2. Some hens had extremely low or high values compared with the mean for some behaviors. During the physical separation, Hen 6 showed a very low movement frequency of 3 toward her chicks compared with the mean value of 17 shown by the other hens, and this same hen displayed a sitting frequency of 10 compared with the mean of other hens that sat down just once. Pacing frequency during the visual separation also varied substantially, with Hen 1 pacing more than 50 times while Hen 4 and 6 paced about 35 times. During visual separation, the frequency of sitting behavior was very low. Hen 5 sat only once during visual separation, while the other five hens did not sit at all during the 10-min period. These variations are evident as outliers in the box plots in Figures 3–7.

There was no difference in the frequencies of eight out of the nine behavior between the two separation types; the movement of the hen toward her chicks (z = −0.315, *P* = 0.752, Figure 3A), movement away from her chicks (z = −0.314, *P* = 0.753, Figure 3B), preening (z = −0.420, *P* = 0.674, Figure 4A), body shaking (z = −0.552, *P* = 0.581, Figure 4B), defecation (z = −0.276, *P* = 0.783, Figure 5A), sitting (z = −1.625, *P* = 0.104, Figure 5B), pecking (z = −0.365, *P* = 0.715, Figure 6A), and jumping (z = −1.461, *P* = 0.144, Figure 6B). However, the frequency of pacing (z = −2.201, *P* = 0.028, Figure 7) was greater when the hens were separated from their chicks visually than when they were separated physically.

**Figure 8:**
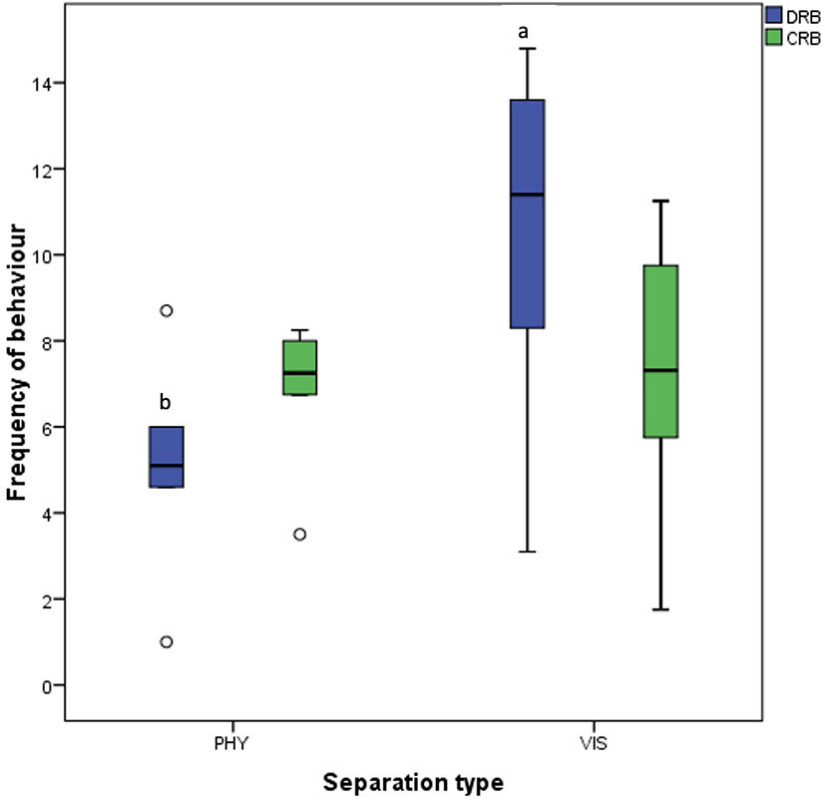
Frequency of discomfort-related (DRB) and comfort-related (CRB) behaviors of the mother hens when subjected to 10-minute physical (PHY) or visual (VIS) separation from their chicks. ^ab^Means differ at *P* < 0.05 for discomfort-related behaviors.

For the grouping of the behaviors, there was a greater [*t*_(5)_ = −2.717, *P* = 0.042] display of discomfort-related behavior in hens during visual separation than in physical separation, but no difference [*t*_(5)_ = −0.231, *P* = 0.827] in comfort-related behavior in hens subjected to physical or visual separation (Figure 8).

Finally, the actual values of the eye temperature, heart rate, and blood glucose before and after each separation and the changes (after separation minus before separation) in eye temperature, heart rate, and blood glucose were similar (*P* > 0.05) in both separation types (Table 2).

**Table 2:** Physiological responses, actual values, and changes (after separation minus before separation) of Nigerian indigenous hens to physical and visual separation from their chicks.

	**Eye temperature**	**Heart rate**	**Blood glucose**
	**(°C)**	**(beats/minute)**	**(g/dL)**
**Physical separation**			
Before	37.27 ± 0.03	176.18 ± 0.17	203.88 ± 0.17
After	37.35 ± 0.25	176.52 ± 3.03	188.88 ± 6.02
Changes	0.08 ± 0.23	0.33 ± 3.07	−15.00 ± 5.98
**Visual separation**			
Before	37.53 ± 0.05	175.55 ± 0.73	185.40 ± 0.27
After	37.50 ± 0.33	174.38 ± 6.57	178.98 ± 5.05
Changes	−0.03 ± 0.31	−1.17 ± 6.60	−6.42 ± 4.90

Values are means ± SE.

## Discussion

The low number of hens in this study constrained us from making a definite conclusion, but we discuss the implications of these preliminary results for the management and welfare implications of the indigenous chickens. This exploratory study compared the behaviors of Nigerian indigenous hens when separated physically and visually from their chicks for 10 min on the 8^th^ and 12^th^ days of post-hatch, respectively. The tests on the six hen-chick pairs were conducted on different days to reduce the level of stress and the possibility of a masking effect of one separation type on the other one.

The experiment did not follow a cross-over design. Hence, the possibility of a confounding effect of the chicks' age with the type of separation cannot be totally ruled out. Nevertheless, there were only 3 days between the two tests and Edgar et al. (3) observed no effect of counterbalancing hens on their behavioral and physiological responses to their 3 to 4-week-old chicks' distress conditions.

These two separation methods were used because they were the most commonly occurring scenarios in the life of a scavenging mother hen and chicks. The separation lasted for 10 min to understand the hens' immediate behavior when separated from their chicks. We adopted a short-term separation based on previous reports that long-term (4-h) separation of 4-day-old chicks resulted in the chicks not being able to identify their mothers afterward (18). Also, the removal of 3-day-old chicks from their mothers caused a fast reduction in maternal responsiveness. The hen stopped clucking completely 4 days after withdrawal, but 56% of them still made tidbits calls, which decreased to 33% after a week of chick removal (19).

The low number of broody hens (20%) could be attributed to the adoption of a natural brooding method in this study, where eggs were left in the nest boxes and the hens were exposed to natural conditions (12L:12D). However, inducing broodiness by increasing the daylight to 16L in addition to the provision of eggs in the nest box resulted in 46.7% of brooding in the Silkie and Wyandotte hens (11, 20). It would be interesting to investigate if the induction of broodiness works in tropical breeds as reported in temperate breeds.

After hatching, hens become aggressive toward intruders to protect their chicks, a behavior known as maternal aggression. The intruders could be humans or predators. The mother hen displays maternal aggression for as long as the chicks are still under her care, especially in the first few weeks after hatching. The hens in the current study seemed to find the physical separation type less stressful, probably because they could see their chicks and communicate with them. Our result agrees with the report of Madec et al. (21) that hens experience less stress if they have visual contact with their chicks but could not come in contact with them physically. This type of separation could be adopted by rural poultry farmers as a means of a gradual weaning process instead of the abrupt weaning of chicks from their mothers. The farmers can subject the mother and chicks to physical separation a few times a day for several days before the chicks are finally weaned. By doing this, the chicks would have gotten used to being separated from their mothers, and when finally weaned, they would not find the weaning process stressful. However, this proposed welfare-friendly weaning process of chicks requires investigation to determine how often, at what age, and how many days are required for the chicks to become accustomed to this process.

On the other hand, the mother hens displayed greater discomfort-related behaviors (pacing, movement toward the chicks, body shaking, defecation, and jumping) when visually separated from their chicks. This implies that the mother hens are more distressed by this type of separation because they could only communicate with their chicks but not see each other.

Some hens hatch a good number of chicks, but within the first week of life, most of the chicks have been predated upon, while some are able to keep all or most of their chicks for survival. Since these indigenous chickens are reared by poor rural farmers under a scavenging system, there is a need to identify and select breeds with high maternal care and aggression. For greater survivability of the chicks in the natural environment, the aggressiveness of the hens is a determining factor. For this study, we discovered some individual differences in the hens' behavior that could indicate their maternal styles.

A preliminary observation of the recorded behaviors of the hens was made and used to develop the ethogram reported in this study, as we could not find any existing ethogram for this kind of test in the literature. We observed some individual differences between the hens. The differences observed could be related to the differences in the number of chicks reared by each hen (the number of chicks ranged from three to seven in this study). There could be the possibility of maternal aggression corresponding with the hen's having a lower or greater number of chicks to protect. This would be a research idea for future studies. For instance, Hen 1 paced more than 50 times while Hens 4 and 6 paced about 35 times. It could be speculated that these three hens, which showed high pacing, might have a better mothering ability and were not ready to give up in their attempt to reunite with their chicks. Selection and multiplying hens with this trait could be beneficial for outdoor production because an increased maternal aggression confers on them the ability to protect their chicks when faced with real-time predators. Some mother hens go to the extreme of attacking predators such as hawks or snakes to protect their chicks (personal observation).

In the six hen-chicks pairs used in this study, eight of the behaviors monitored are performed in a similar pattern by hens in both separation types which implies that they reacted similarly to being separated from their chicks, irrespective of whether it is a physical or visual separation. However, the pacing frequency or frequency of discomfort-related behavior (pacing, movement toward chicks, jumping, defecation, and body shaking) is higher in hens during visual separation, which might suggest that the hens perceived the visual separation to be more stressful. During the visual separation, the hen and chicks could only communicate through vocalization. Increased pacing has been associated with restlessness. Animals tend to show an increased pacing when they are under stress or unable to express species-specific behaviors (22, 23). Pacing is considered a stereotypic behavior (24–26) when it is performed for no apparent reason. The pacing behavior observed in this study is a way for the hen to express her discomfort at being separated visually, as she was looking for all means possible to reunite with her chicks.

There was the possibility that the hens were more stressed in the visual separation test. However, this was not supported by physiological responses. The reason for the lack of a difference in the stress responses between the physical and visual separation types could be attributed to the low number of hens used in this study or the short duration of the separation period (10-min). The choice of 10-min was intentional to reduce the distress experienced by the hens to the barest minimum.

One of the limitations of this study was that we failed to monitor the behavior and physiological responses of the chicks during the separation period. During the separation period, the chicks made distress calls in both separation types (personal observation). Wauters and Richard-Yris (27) also reported that chicks began to emit distress calls when they lost visual contact with their mothers (27). However, there may be a possibility of the chicks emitting a highly intense type of distress call depending on how stressful they might have perceived the two separation types. If the chicks perceive the visual separation to be more stressful, they could communicate this to their mothers, which might arouse her emotions to become more restless by increased pacing. Hens understand the distress conditions of their chicks (3). Mother hens modify their behaviors based on the signals from their chicks. In a recent study where chicks of two age categories (5–6 weeks old and 5–7 days old) were isolated from conspecifics for 5 min, the distress call made by the 5–7 days old chicks were of a greater peak frequency, and the vocalization characteristics in both chick groups were positively correlated with changes in surface body temperatures (28). Further studies are therefore required to investigate the effects of the two separation types on the chick's behavior and physiology.

## Conclusion

This is an exploratory study on the behavior and physiological responses of Nigerian indigenous hens to visual and physical separations from their chicks. The behaviors of the hens are similar in the physical and visual separations, but hens reacted to visual separation from chicks by increasing pacing. Overall, hens displayed greater discomfort-related behaviors during visual separation than physical separation from their chicks. Physiological responses are similar in both separation types. Concrete conclusions cannot be made due to the low sample size used in this study. However, the findings from the study could serve as insights for future research on these chickens. Further studies should investigate the behavior, physiology, and distress call characteristics of the chicks under these two separation types. Future studies are needed to compare the responses of different chicken breeds or Nigerian indigenous chicken ecotypes to these separation types. Finally, it would be interesting to know whether maternal aggression has a positive influence on the chicks' survival and welfare.

## Data availability statement

The raw data supporting the conclusions of this article will be made available by the authors, without undue reservation.

## Ethics statement

The procedure for the experiment was approved by Animal Care and Use Committee of the College of Animal Science and Livestock Production of the Federal University of Agriculture, Abeokuta, Ogun State, Nigeria.

## Author contributions

OI was involved in conceptualization, investigation, data collection, data analysis, and manuscript writing. SD and KO were involved in the investigation and manuscript writing. OPO, OEO, OF, and VO were involved in the investigation and data collection. All authors contributed to the article and approved the submitted version.

## Conflict of interest

The authors declare that the research was conducted in the absence of any commercial or financial relationships that could be construed as a potential conflict of interest.

## Publisher's note

All claims expressed in this article are solely those of the authors and do not necessarily represent those of their affiliated organizations, or those of the publisher, the editors and the reviewers. Any product that may be evaluated in this article, or claim that may be made by its manufacturer, is not guaranteed or endorsed by the publisher.
